# *QuickStats:* Percentage* of Adults Aged ≥18 Years with Diagnosed Chronic Obstructive Pulmonary Disease,^†^ by Urbanization Level^§^ and Age Group — National Health Interview Survey, United States, 2019^¶^

**DOI:** 10.15585/mmwr.mm7026a3

**Published:** 2021-07-02

**Figure:**
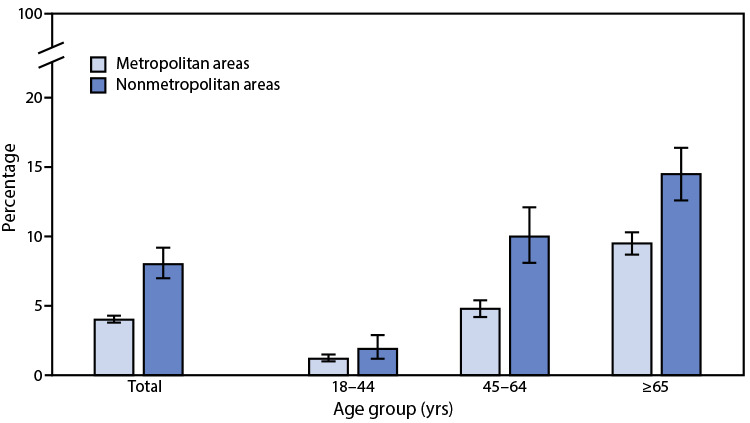
In 2019, the percentage of adults aged ≥18 years with diagnosed chronic obstructive pulmonary disease (COPD) was higher among those living in nonmetropolitan areas (8.0%) than among those living in metropolitan areas (4.0%). Percentages were higher in nonmetropolitan areas for adults aged 45–64 years (10.0% versus 4.8%) and aged ≥65 years (14.5% versus 9.5%), but the difference by urbanization level was not statistically significant for adults aged 18–44 years (1.9% versus 1.2%). The prevalence of diagnosed COPD increased with age in both nonmetropolitan and metropolitan areas.

**Source:** National Center for Health Statistics, National Health Interview Survey, 2019. https://www.cdc.gov/nchs/nhis.htm

